# ResNet18 facial feature extraction algorithm improved based on hybrid domain attention mechanism

**DOI:** 10.1371/journal.pone.0319921

**Published:** 2025-03-19

**Authors:** Yingying Mei

**Affiliations:** Faculty of Intelligent Transportation, Anhui Sanlian University, Hefei, China; Thapar Institute of Engineering and Technology: Thapar Institute of Engineering and Technology (Deemed to be University), INDIA

## Abstract

In the research of face recognition technology, the traditional methods usually show poor recognition accuracy and insufficient generalization ability when faced with complex scenes such as lighting changes, posture changes and skin color diversity. To solve these problems, based on the improvement of adaptive boosting to improve the accuracy of face detection, the study proposes a residual network 18-layer face feature extraction algorithm based on hybrid domain attention mechanism algorithm. The study introduces channel-domain and spatial-domain attention mechanism to enhance the extraction of face image features. The outcomes indicated that the recognition accuracy of the proposed method on multiple face image datasets, labeled field face datasets, and celebrity facial attribute datasets exceeded 98.34% and reached up to 99.64%, which was better than the current state-of-the-art methods. After combining channel and spatial attention mechanism, the false detection rate was as low as 2.50%, which was lower than the false detection rate of other methods. In addition to enhancing face recognition’s robustness and accuracy, the work offers fresh concepts and resources for face recognition’s potential uses in intricate scenarios in the future.

## 1. Introduction

Under the trend of rapid development of global information technology, face recognition technology has become an important part of the field of biometrics, which is widely used in many fields such as security verification, access control, and monitoring system. Face recognition technology is a very practical and user-friendly non-contact method of identification authentication that greatly simplifies people’s life [1]. The core of FR technology lies in realizing the automatic identification of individuals by analyzing the visual features of faces [2]. FR has changed from its original geometric feature analysis to sophisticated feature learning based on deep learning (DL) since the 1960s as a result of advancements in computer vision and pattern recognition technologies [3]. Early FR techniques are based on manually extracting face features such as the positional relationship of eyes, nose and mouth. It has a certain degree of recognition capability. However, when faced with external factors such as lighting changes, expression changes, posture changes, etc., the early technology shows poor adaptability [4]. The development of face recognition technology, which can automatically learn the complex elements of an image to dramatically increase recognition robustness and accuracy, has been greatly aided by the advent of DL technology [5,6]. However, existing DL-based face recognition techniques still face many challenges in practical applications. For instance, a lot of methods still require a lot of labeled data for training, which is challenging to come by in real-world settings. In addition, most face recognition techniques suffer from insufficient generalization ability when facing faces with different skin colors (SCs), ages and lighting conditions. Due to this, the study initially suggests a face identification technique based on enhanced SC segmentation and adaptive boosting (AdaBoost). The article suggests a face feature extraction approach based on face detection (FD) and then employing the hybrid domain’s attention mechanism to enhance the residual network 18-layers (ResNet18) network. The study aims to construct a face recognition technique with high recognition accuracy (RA) and strong robustness by the proposed FD method and face feature extraction algorithm, and to improve the adaptability of the face recognition technique.

The main contribution of the research is to propose an improved ResNet18 network structure based on mixed-domain attention mechanism, which significantly improves the accuracy and robustness of feature extraction by combining channel-domain (CD) and spatial-domain (SD) attention mechanism. It provides a higher guarantee for the performance of face recognition technology in complex application scenarios. In addition, the AdaBoost algorithm is improved and combined with SC segmentation technology, which effectively reduces the false detection rate (FDR) and the missing detection rate, making the method more suitable for actual authentication and security monitoring applications.

The research contains four sections. The first section provides a comprehensive analysis of the existing face recognition techniques. The second section provides a detailed introduction and description of the FD method and face feature extraction algorithm proposed in the research. Through numerous studies, the third section demonstrates the superiority and efficacy of the research content. The article’s shortcomings are highlighted, the study’s overall findings are summarized, and recommendations for additional research are given in the fourth section.

## 2. Related work

Face recognition technology is a key area of biometric recognition that has advanced rapidly in the last many years. Driven by DL technology, the accuracy and application scope of face recognition technology have been significantly improved. At present, face recognition is widely used in many fields such as security monitoring, mobile payment, identity verification, etc., which greatly improves the convenience of life and work [7]. Therefore, many scholars around the world have conducted research on face recognition technology. To cope with the recognition needs in complex environments and diverse application scenarios, researchers continue to propose new methods to improve the robustness and accuracy of face recognition technology. Aiming at the problem of self-occlusion and perception difference in near-infrared-visible heterogeneous face recognition, R. He et al. proposed a high-resolution heterogeneous face synthesis method. By introducing texture repair components and pose correction components, this method reduced spectral and pose differences and significantly improved the accuracy of heterogeneous face recognition [8]. Although this method is effective for cross-spectral recognition, its compensation mechanism is contingent upon specific spectral conditions, which presents a challenge for the promotion of its use in multi-illumination or extreme environments. The combined face alignment and 3D face reconstruction method proposed by F. Liu et al. realized the combination of 2D alignment and 3D reconstruction through iterative cascade regression, which greatly improved the accuracy of face recognition across posture and expression changes [9]. However, the 3D face reconstruction method is highly data dependent, which makes it less applicable to scenes with limited data acquisition, and increases the complexity and computational cost of the algorithm. In response to racial bias in face recognition, J. Muhammad et al. built the CASIA-Face-Africa database, which specifically collected and tagged large-scale African face images. This study provided the data basis for the study of racial bias, and realized the benchmarking and optimization of the performance of existing algorithms [10]. Nevertheless, it is challenging for such databases to comprehensively encompass the attributes of heterogeneous ethnic groups. Moreover, there are still constraints in the capacity of models to accommodate the generalization of diverse ethnicities. To improve the robustness of face recognition in real scenes, H.I. Kim et al proposed a depth feature alignment framework guided by face shape, which enhanced the model’s performance in the case of facial dislocation through pixel-level and feature-level alignment [11]. Although this method has achieved good results in facial dislocation scenes, its adaptability to high occlusion and complex background scenes needs further investigation.

In addition, to improve the robustness of the model and optimize the tagging task, A. Amirkhani et al. proposed the multi-teacher knowledge distillation (KD) paradigm, which used multiple expert teacher models. This could guide the student model to increase its robustness and accuracy under different conditions and achieve significant improvements in semantic segmentation tasks [12]. At the same time, A. Banitalebi-Dehkordi et al proposed an energy-based course learning method, which improved the reliability of pseudo-labels by gradually adapting to the model and using SD mixing and KD strategies. This study improved the performance of the model in unsupervised domain adaptation [13]. While these methods have advantages in improving task-specific model performance, their task-specific design limits their breadth of application in multimodal face recognition scenarios.

For face and expression recognition in complex environments, A. Sepas-Moghaddam et al. proposed the CapsField framework. It combined convolutional neural network (CNN) with capsule network, and used dynamic route learning to realize hierarchical relationship between capsules. Therefore, this study improved the effect of face and expression recognition in complex environments [14]. However, CapsField still has the possibility of declining RA when dealing with rapidly dynamic changing environments. The template inversion attack defense method proposed by H. Otroshi Shahreza et al. realized resistance to template inversion attacks by training high-resolution face reconstruction models on synthetic data. However, its security performance in practical applications needed further verification, and the generality of the method in the face of different template structures was still insufficient [15]. M.-T. Chiu et al. proposed a new segmentation guidance network combining RGB-D recognition branch and auxiliary segmentation mask to solve the overfitting problem caused by the lack of public 3D face dataset and small sample size. The network used semantic segmentation to improve the positioning accuracy of the face region, thus improving the recognition effect [16]. However, there are limitations in the actual acquisition of RGB-D data, which affects the wide application of this method. Aiming at the problem of intra-class variation in face recognition from near infrared to visible light, W. Hu et al. proposed a method of domain difference elimination and mean face representation learning, which improved the performance of cross-mode recognition through class-specific domain difference elimination and cross-mode mean matching [17]. Although the method is effective under certain spectral conditions, its adaptability in multi-device and cross-environment applications still needs to be improved.

In general, although the existing research has made some achievements in improving the performance of face recognition, there are still limitations of insufficient generalization ability and robustness, especially in the RA under multiple skin tones, complex lighting, expression changes, and occlusion conditions. Most of the existing methods are based on specific data sets or specific environments, which makes it difficult to meet the needs of diverse scenarios in practical applications. In addition, the effect of deep-level feature extraction is still limited, which limits the performance of face recognition in practical applications. Therefore, the study proposes a FD method with face feature extraction algorithm. The study improves the AdaBoost algorithm and combines it with SC segmentation to achieve FD, and improves the ResNet18 network structure by using the hybrid domain-based attention mechanism. This is creative and improves the network’s capacity to extract important face features.

## 3. Methods and materials

The AdaBoost algorithm is first introduced in the study. Second, a FD technique built on top of the enhanced AdaBoost algorithm is suggested. The work creates an attention mechanism module that integrates CD and SD, and it further refines the ResNet18 network based on the output findings of the FD approach. Finally, the design of face feature extraction algorithm is carried out on the basis of the improved ResNet18 network.

### 3.1. Face detection method based on improved AdaBoost

In face recognition technique, FD is the prerequisite for face feature extraction. FD, as the first step, is responsible for locating the face region in an image or video frame, which in turn is used to obtain features such as color, texture, geometric shape, depth information, etc. by face feature extraction algorithm. Therefore, to address the existing drawbacks of face recognition techniques, the study first proposes a FD method based on improved AdaBoost with SC segmentation. AdaBoost algorithm is a widely used integrated learning algorithm, which is characterized by high accuracy, real-time and easy implementation in face recognition [18]. However, AdaBoost relies on the quality of the weak classifiers (WCs) and suffers from the risk of overfitting. Moreover, as the WCs rises, the training and detection process of AdaBoost may be computationally intensive [19,20]. Therefore, AdaBoost needs to be improved.

**Fig 1:**
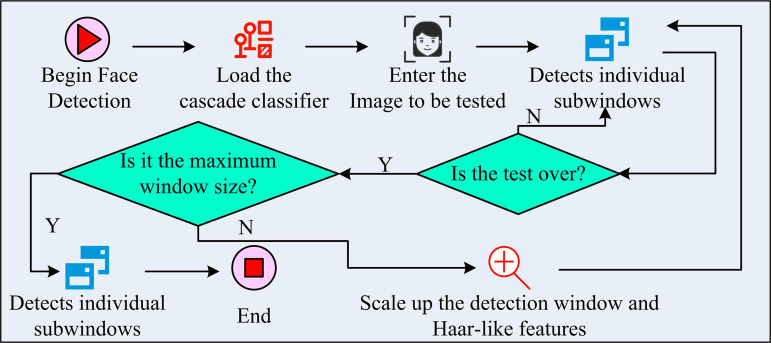
Face detection process based on AdaBoost algorithm.

The first part of the research examines the three primary phases involved in implementing the AdaBoost algorithm. The training samples are assigned the same beginning weights in the first phase. In the second stage, the weights of correctly classified samples (CSs) are decreased and the weights of misclassified samples (MCSs) are increased as WCs are trained based on the weights in each iteration. The third step combines all the WCs weighted according to their classification effects into strong classifiers. Among them, the effective classifiers have higher weights and have more influence on the final decision. Subsequently, the study analyzes the flow of FD using AdaBoost algorithm, as shown in Fig 1.

**Fig 2:**
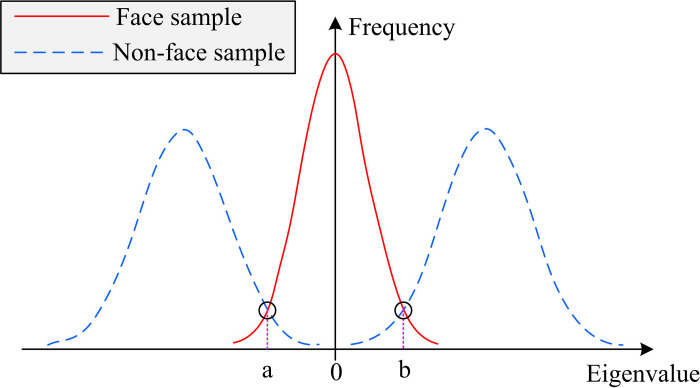
Distribution of face and non-face features in Haar-like features.

In Fig 1, a cascade classifier needs to be loaded first after FD starts. Since face recognition is a complex classification problem, WCs are less effective when used alone. Therefore, it is studied to linearly combine multiple WCs and utilize their synergistic effect to form a cascade classifier. As the cascade goes deeper, the accuracy of classification detection is gradually improved. Subsequently, the image to be detected is input. During the detection process, the study replaces the operation of scaling the entire image by resizing the detection window to reduce the amount of computation. Once a face is detected, the relevant window will be further verified by cascading strong classifiers until final confirmation. Additionally, a merge threshold is utilized to merge the close rectangular boxes to establish the final face region, hence resolving the issue that the same face may be detected more than once. The study uses Haar-like features to train the classifiers in AdaBoost. However, the face samples in Haar-like features and non-face samples show a clear difference in the distribution of eigenvalues, as shown in Fig 2.

In Fig 2a and b are the two threshold points where faces and non-faces possess significant differences. The frequency of non-facial features is significantly higher than that of face features when the feature value is less than an or greater than b. When the feature value is within the interval of a and b, the frequency of face features is much higher than that of non-face features. When using the traditional AdaBoost algorithm for FD, if only a single threshold is used to decide whether the feature value represents a face or not, some features that do not actually belong to a face will be misclassified due to the overly loose threshold setting. This operation not only increases the FDR, but also may reduce the accuracy of detection. In addition, this approach may pose a risk in security-sensitive applications because it may allow unauthorized individuals to pass through the recognition system. Therefore, the study improves the single threshold setting in the traditional AdaBoost algorithm and utilizes the dual threshold setting method to enhance the performance of FD. For the Haar-like features in the face samples, the difference $\Delta f\left(x\right)$ between the frequency of occurrence of the face features and the frequency of occurrence of the non-face features is first calculated, as shown in Eq (1).


$$\Delta f\left(x\right)=-f_{2}\left(x\right)+f_{1}\left(x\right)$$ (1)

In Eq (1), $f_{1}\left(x\right)$ is face feature frequency map. $f_{2}\left(x\right)$ denotes the non-face feature frequency map. At this point, search for the point where the feature difference between the face and the non-face is most obvious, i.e., the point with the largest $\Delta f\left(x\right)$ value, $x_{0}$. Then search for the threshold endpoints on both sides of $x_{0}$ to find the eigenvalue point that makes the $\Delta f\left(x\right)$ value 0. If no such point can be found, two threshold points $x_{1}$ & $x_{2}$ are determined where the value of $\Delta f\left(x\right)$ is close to 0. The classification formula for improving the WC using the dual threshold setting method is shown in Eq (2).


$$h_{j}\left(x\right)= \{\begin{array}{l} 1,ifx_{1}\leq f_{j}\left(x\right)\leq x_{2}\\ 0,\text{otherwise} \end{array}$$ (2)

In Eq (2), $h_{j}\left(x\right)$ denotes the classification result of the *j* th WC for the sample. $f_{j}\left(x\right)$ denotes the eigenvalue of the *j* th Haar-like feature. In addition to the ability of the dual threshold setting to improve the differentiation accuracy between faces and non-faces, it is also able to reduce the multiplication operation because the CPU performs addition and subtraction much faster than multiplication. This reduces the time cost of calculating the error and improves the training efficiency. The weights of all samples are updated, the weights of correctly CSs are decreased, and the weights of MCSs are increased when a WC is trained using the conventional AdaBoost method. This may lead to overweighting of difficult to classify samples, which in turn causes weight imbalance and affects the performance of the WC [21,22]. Therefore the study improves the process of weight updating. First the study will calculate two metrics. At this point, the judgment is made using Eq (3) as follows.


ω≥μ+3σ (3)

**Fig 3:**
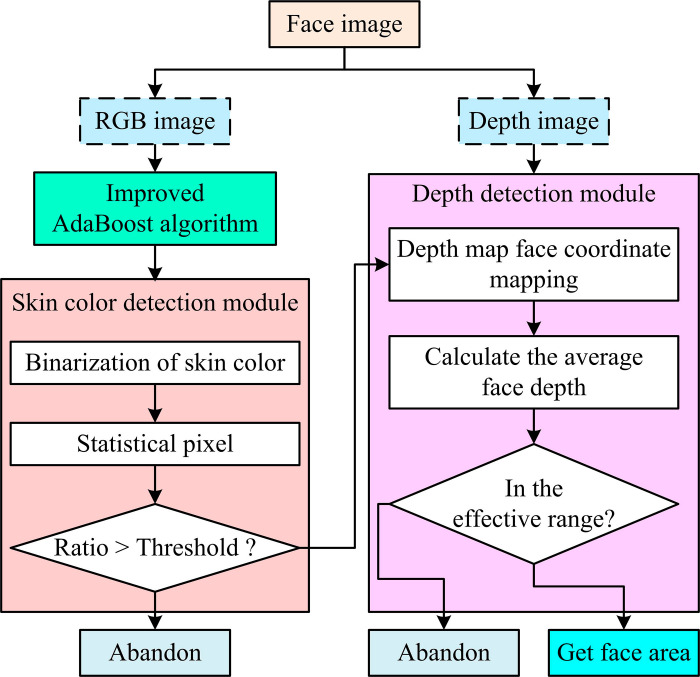
Flow of complete face detection method.

In Eq (3), *ω* denotes the sample weights. *μ* denotes the mean. *σ* denotes the standard deviation. If Eq (3) holds for a certain weight, the weight is kept constant in the iteration to avoid the effect of extreme values. In addition, improvements include subdividing the MCSs and adjusting the weight updates. That is, the MCSs are divided into positive sample misclassification and negative sample misclassification, and the weight update rule is adjusted according to the misclassification rate. To further enhance the accuracy of FD, the study also combines the SC detection module with the depth detection module on the basis of the improved AdaBoost algorithm (IABA) to form a complete FD method. The specific flow is shown in Fig 3.

In Fig 3, the proposed detection method divides the FI into RGB image (RGBI) and depth image (DI). For the RGBIs, preliminary detection is first performed using the IABA. Subsequently, the study introduces a SC detection module to secondary screen the initially detected face regions with a SC similarity calculation method based on Gaussian SC model. The SC similarity is calculated for each pixel point in the RGBI by binarizing the similarity values of these pixel points in the SC detection process. Then a threshold is set to ensure that only regions with a percentage of SC pixel points above a certain percentage are recognized as valid face regions. For the DI, the study then utilizes the distance information of each pixel point by mapping the specific location of the face region in the DI and calculating its depth average value. If this average value lies within the preset valid range, the successful detection is confirmed and the region is retained, otherwise the region is discarded. The proposed FD method integrates SC information, AdaBoost detection, image alignment and depth information to improve the accuracy and robustness of FD.

### 3.2. Face feature extraction algorithm based on improved resnet18

**Fig 4:**
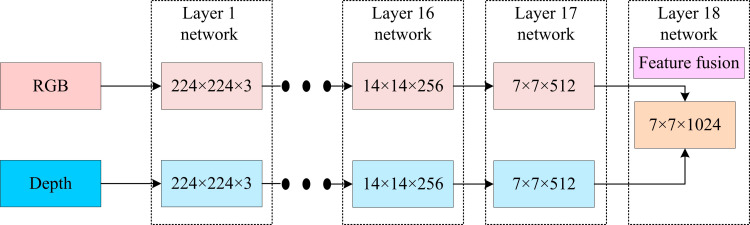
Fusion process of two ResNet18 networks for extracting features.

After completing FD, more detailed face feature extraction can be performed, so the study further proceeds with the design of face feature extraction algorithm. In the field of face feature extraction, CNN, as a key technique of deep modeling, is extremely useful in enhancing the accuracy of face feature extraction [23]. However, in CNN-based face feature extraction methods, multi-layer convolution, pooling, and full connectivity do not achieve accurate extraction of face edges and textures, etc [24]. While residual network ResNet18, a variant of deep CNN, improves the network structure by introducing residual block (RB). It is able to effectively transfer gradients and reduce gradient vanishing during the processing of more complex image data [25,26]. To achieve effective feature extraction of FIs, the study used two independent ResNet18 networks to special extract and fuse the RGBI containing color information with the DI that provides three-dimensional information related to distance, respectively. The fusion process can be shown in Fig 4.

In Fig 4, the two ResNet18 networks perform fusion of extracted features at the last layer after 17 layers of image features have been extracted for the RGBI and DI respectively. Effective FI feature extraction is achieved by joining the information from both images together. However, image feature extraction from only two ResNet18 networks does not guarantee the effectiveness of the algorithm [27]. Therefore, this study introduces a hybrid domain attention mechanism in the ResNet18 framework, including the attention mechanism in the CD and the SD. The channel attention mechanism gives different weights to each channel in the feature graph to emphasize features relevant to the recognition task and reduce interference. The process involves maximum pooling and average pooling operations to obtain a global feature representation, and generates attention weights through convolution and activation functions. The spatial attention mechanism focuses on the importance of different spatial locations in the feature map, generates spatial feature representations through maximum pooling and average pooling operations, and uses convolution to compute the spatial attention coefficient to enhance feature locations that contribute to classification. Embedded in each residual module of ResNet18, the mechanism applies channels and spatial attention modules in series to enable the network to more accurately capture key facial features, thereby improving the accuracy and robustness of recognition. The study sets the dimension of the input feature image as G×H×W. Among them, *G* is the channels of the image, *H* is the height of the image, and *W* is the width of the image. The expression formula of the feature vector obtained after CD based attention mechanism extraction can be shown in Eq (4).


$$P_{\alpha }^{G}=Conv_{1\times 1}(P_{\max}^{G}⊕P_{avg}^{G})$$ (4)

In Eq (4), $P_{\alpha }^{G}$ is the feature vector of the image of dimension G×1×1 size. $Conv_{1\times 1}$ is 1 × 1 convolution. $P_{\max}^{G}$ is the feature vector obtained by maximum pooling for an image of dimension G×1×1 size. $P_{avg}^{G}$ denotes the feature vector obtained by average pooling for an image of dimension G×1×1 size.  ⊕  denotes summing by bits. When all the feature vectors are convolved by 2 times and ReLU convolution, its combined with Sigmoid activation function to get the attention coefficient of the image channel [28–30]. Eq (5) provides the specific calculating formula.


$$\gamma_{G}=\vartheta_{S}(W_{1}(\vartheta_{R}(W_{0}(P_{\alpha }^{G}))))$$ (5)

**Fig 5:**
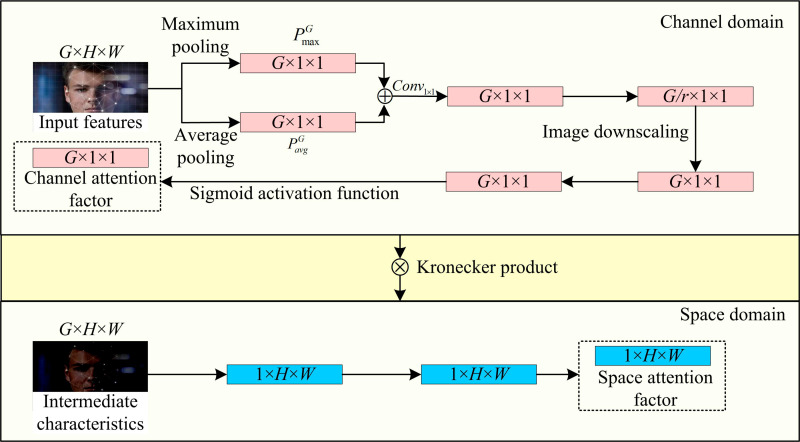
Structure of attention mechanisms based on channel and SDs.

In Eq (5), $\gamma_{G}$ is the attention coefficient of the image channel. $\vartheta_{R}$ is the ReLU function. $\vartheta_{S}$ is the Sigmoid function. $W_{0}$ and $W_{1}$ denote the parameters of the two $Conv_{1\times 1}$. The CD and SD based attention module can be shown in Fig 5.

In Fig 5, in the attention mechanism of CD, the network assigns different weights to each channel, i.e., to a set of features in each feature map. The network is able to reduce the influence of less relevant information while emphasizing those that are more critical for face recognition by allocating various weights to different channels. SD, on the other hand, mainly learns the correlation of different spatial locations in the feature image in an attempt to extract the spatial locations of features with higher importance in the feature image. Therefore, the image feature compression method used in SD is different from CD. It is assumed that there exists an intermediate feature vector *P*^′^ adjusted by the CD based on the attention mechanism. Its processed by the SD, a feature image based on the spatial attention coefficient is obtained, and the specific calculation formula can be shown in Eq (6).


$$\gamma_{s}=\vartheta_{s}(Conv_{3\times 3}(Conv_{1\times 1}(P^{\prime })))$$ (6)

**Fig 6:**
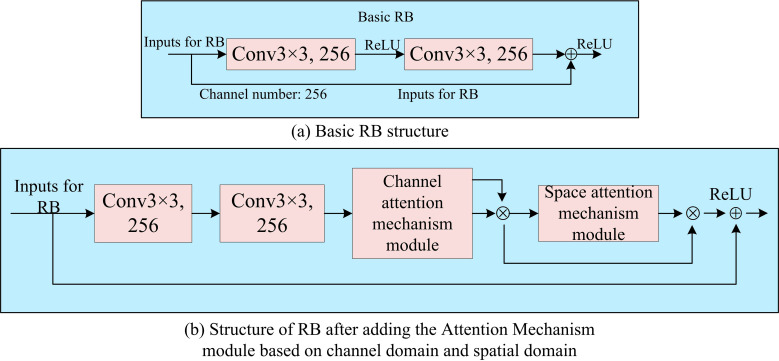
Comparison of the structure of the basic RB and the RB with the addition of the attention mechanism module.

In Eq (6), $\gamma_{s}$ denotes the attention coefficient in image space. $Conv_{3\times 3}$ denotes the 3 × 3 convolution. When $\gamma_{s}$ is determined, *P*^′^ is updated based on the value of $\gamma_{s}$ and multiplied bitwise based on the Kronecker product [31,32]. On this basis, the study further introduces an attention mechanism module based on CD and SD in each RB of ResNet18 network. Furthermore, the CD and the SD are connected in series as one. The specific RB structure can be shown in Fig 6.

**Fig 7:**
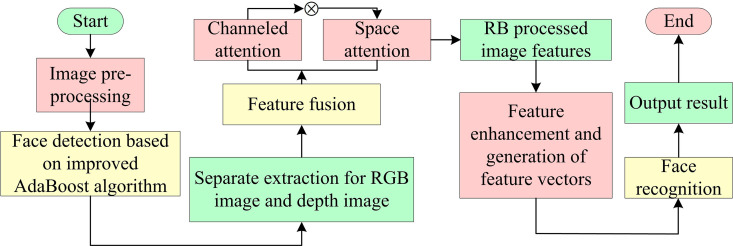
Extraction process of face feature extraction algorithm based on improved ResNet18.

In Fig 6(a) and (b), after adding the attention mechanism module based on CD and SD in RB, the network is able to capture the information that is useful for the recognition task more accurately and improve the quality of the extracted image features when processing the image features. It is assumed that the input features of RB have 256 channels. First, these input features are subjected to two 3 × 3 size convolution operations to extract the local information in the input features. After that, the extracted features are passed to the attention mechanism module of the CD. Furthermore, the weight of each channel in feature extraction is correlated with the attention coefficient, which is determined based on the significance of each channel. The computed CD attention coefficients are multiplied point by point with the input features at the element level. After CD optimization the features are further passed to the attention mechanism module of the SD. The importance of features at each spatial location is analyzed and an attention coefficient is assigned to each location. Finally, by adding the input features with the features optimized by convolution and attention mechanism, the original information is retained while more useful features are introduced. Combining the above, the extraction flow of the face feature extraction algorithm based on improved ResNet18 proposed in the study can be shown in Fig 7.

The study’s suggested facial feature extraction algorithm preprocesses the input FI shown Fig 7. Based on the outcomes found by the upgraded AdaBoost algorithm in the RGBI and DI, respectively, the face characteristics are also extracted. Features from RGB and DIs are extracted using two different ResNet18 networks, and the fusion technique combines the features from both modalities. Secondly, an attention mechanism based on CD and SD is introduced to improve the RB of ResNet18 network and image feature extraction and output.

## 4. Results

To verify the effectiveness and superiority of the FD method based on IABA and face feature extraction algorithm based on improved ResNet18 network proposed in the study, the study conducts experiments on both of them respectively. The main experiments include ablation experiments as well as comparison experiments.

### 4.1. Performance validation and analysis of face detection methods

Prior to the start of the experiment, the study constructs a diverse face image dataset (MFID) to compensate for the lack of diversity in existing publicly available datasets. The MFID contains approximately 20,000 facial images of different races (Asian, African, Latino, Caucasian, and Middle Eastern), ages (from children to the elderly), and genders, with a balanced sample. In addition, the dataset includes a variety of postures (frontal, lateral, tilted) and expressions (smiling, serious, surprised, etc.) to enhance the model’s adaptability to complex situations. In addition, MFID contains not only traditional RGBIs, but also DIs to accommodate ResNet18 networks. Furthermore, two public and well-known datasets, Labeled Faces in the Wild (LFW) (http://vis-www.cs.umass.edu/lfw/) and the CelebFaces Attributes Dataset (CelebA) (https://mmlab.i.e.,cuhk.edu.hk/projects/CelebA.html), are selected to complement the data. LFW is a widely used, publicly available dataset for facial recognition research, containing more than 13,000 facial images of 5,749 individuals from public sources on the Internet, with a variety of backgrounds, lighting, and camera angles. It is suitable for evaluating the performance of facial recognition systems in complex environments. CelebA is a large dataset of 202,599 images from 10,177 identities used primarily for facial attribute recognition tasks, with 40 labels attached to each image covering a variety of facial features such as smiles, glasses, and beards. The images in the CelebA dataset cover a wide range of lighting, backgrounds, and poses. These two datasets provide rich and diverse training data for face recognition and attribute classification research, helping to improve the performance and robustness of models in various real-world scenarios. Among the three datasets, the MFID dataset is self-generated for the study and has not been publicly released due to the size of the dataset and privacy concerns. Table 1 displays the experiment’s parameter configurations and environment setup.

**Table 1:** Environmental configuration and parameter settings of the experiment.

Configuration item	Details
Framework	Python3.8
GPU	NVIDIA RTX4060Ti (8G)
CPU	Intel® CoreTM i9-13900KF Processor
System	Windows10
Optimizer	Adam optimizer
Memory	32GB
Dataset	MFID, LFW, CelebA
Total training epochs	100 epochs
Batch size	16
Optimization algorithm	Stochastic gradient descent with momentum (Momentum = 0.9)
Initial learning rate	0.01
Learning rate adjustment	Reduce learning rate by 10 times at the 45th and 60th epochs

**Fig 8:**
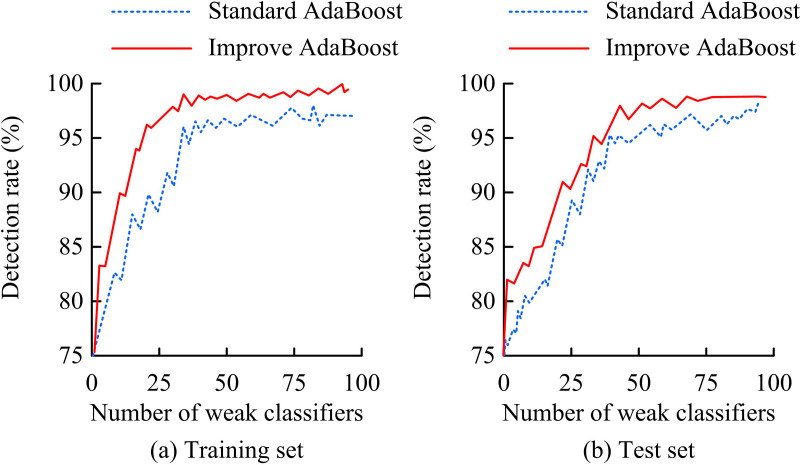
Verification results of improved AdaBoost algorithm.

Based on Table 1, the study further divides the LFW and CelebA datasets into training and test sets in the ratio of 7:3. It is worth noting that all experiments conducted in the study are run 10 times or more, and various random initialization or cross-validation methods are introduced to ensure the reliability and stability of the results. The study first validates the improvement of AdaBoost algorithm on the training and test sets. The results are shown in Fig 8.

In Fig 8(a), in the training set, the IABA rapidly increases the detection rate as the WCs increases. When the WCs reaches about 25, the detection rate is close to 98% and eventually stabilizes above 99.5%. The standard AdaBoost algorithm (SABA), on the other hand, has a lower detection rate under the same conditions, and the detection rate gradually increases to about 96% and then stabilizes. In Fig 8(b), the IABA also shows superior performance in the test set. When the WCs reaches 35, the detection rate has exceeded 95% and eventually stabilized above 97%. The detection rate of the SABA under the same conditions is slightly lower, approaching 95% only when the number of WCs reaches 50, and eventually converging to about 96%. It can be concluded that the IABA has better detection results and stronger generalization ability. In addition, ablation experiments are performed to validate the SC detection module and the depth detection module in the self-constructed MFID dataset. The names of the two modules are set as M1 and M2, and the IABA is set as M0. Table 2 displays the outcomes of the experiment.

**Table 2:** Results of ablation experiment.

Method			Accuracy	Recall	FPR	FNR	IoU
M0	M1	M2					
√	×	×	0.90	0.88	0.08	0.12	0.75
√	√	×	0.93	0.91	0.06	0.09	0.78
√	×	√	0.92	0.90	0.07	0.10	0.77
√	√	√	0.95	0.93	0.04	0.07	0.80

**Fig 9:**
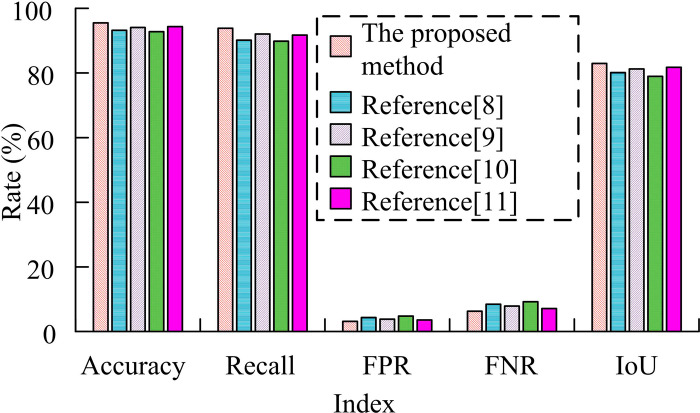
Comparison of face recognition technologies.

In Table 2, FPR, FNR, and IoU denote the FDR, the missed detection rate (MDR), and the intersection and merger ratio, respectively. M0 itself has already shown better detection performance, with an accuracy of 90% and a recall of 88%. After adding M1, the accuracy and recall are increased to 93% and 91%, respectively. Meanwhile, the FDR and MDR decreased, indicating that the SC detection module effectively improves the detection capability of the model. After adding M2, the accuracy and recall are improved to 92% and 90%, respectively. When both M1 and M2 are introduced, the detection performance reaches the best, with an accuracy rate of 95% and a recall rate of 93%. Moreover, the intersection ratio hits 0.80 and the FDR and MDR drop to 0.04 and 0.07, respectively. This suggests that the SC detection and depth detection modules are essential for enhancing the model’s comprehensiveness and accuracy, and that using them together can greatly enhance FD’s overall performance. Finally, the FD technique proposed in the study is compared with the more advanced face recognition techniques mentioned in references [8–10] and [11]. The results are shown in Fig 9.

In Fig 9, the study’s proposed method performs best on all metrics. Specifically, the research method achieves an accuracy of 95.48% and a recall of 93.84%, both of which are better than the methods in other references. It displays that the proposed algorithm is able to detect faces more accurately and miss fewer actually existing faces in the detection process. In addition, the research method has a FDR of 3.16% and a leakage rate of 6.28%, which are lower than other method. It demonstrate its higher detection accuracy and reliability. Ultimately, the research method’s IoU of 82.94% shows that there is more overlap between the detection and real frames. This demonstrates the method’s benefits and efficacy even further in real-world scenarios.

### 4.2. Performance validation and analysis of face feature extraction algorithm

**Fig 10:**
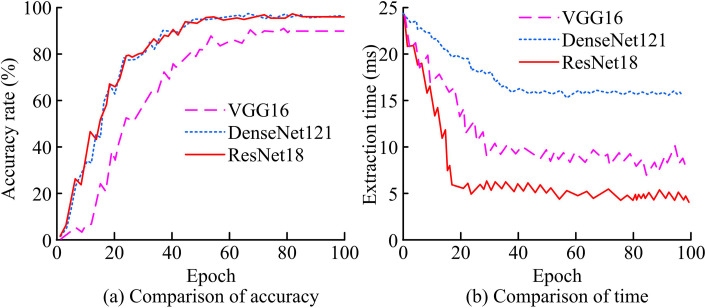
Experimental results of baseline network comparison.

For face feature extraction algorithm, the study first validates the effectiveness of the benchmark network ResNet18 network. Two widely used deep CNN architectures visual geometry group 16-layer network (VGG16) and densely connected convolutional networks 121-layer network (DenseNet121) are chosen as the comparative benchmark networks. Fig 10 presents the outcomes of the experiment.

In Fig 10(a), the accuracy of ResNet18 and DenseNet121 increases rapidly within the first 30 cycles of training, with the two being the first to reach 80%. The accuracy of VGG16 grows relatively slowly, reaching 80% accuracy after 60 iterations. After stabilization, the accuracy of ResNet18, DenseNet121, and VGG16 are 95.34%, 95.17%, and 89.38%, respectively. In Fig 10(b), during the training process, the feature extraction time of VGG16 gradually decreases from 25ms to about 10ms, and that of DenseNet121 is close to 25ms decreasing to about 27ms. In contrast, the feature extraction time of ResNet18 is the lowest in the whole process, gradually decreasing and stabilizing at about 5ms from about 25ms initially. Therefore, the selection of ResNet18 as the benchmark network is more effective. Further, the study conducts ablation experiments. The original ResNet18 is named as N1, channel attention as N2, and spatial attention as N3. Table 3 displays the findings of the experiment.

**Table 3:** Results of ablation experiment.

Method			MFID		LFW		CelebA	
N0	N1	N2	Accuracy	FPR	Accuracy	FPR	Accuracy	FPR
√	×	×	92.30%	5.50%	93.50%	4.20%	90.20%	6.00%
√	√	×	94.10%	4.00%	95.00%	3.00%	92.40%	4.50%
√	×	√	93.80%	4.30%	94.70%	3.20%	91.90%	4.80%
√	√	√	95.50%	3.00%	96.30%	2.50%	93.80%	3.50%

**Fig 11:**
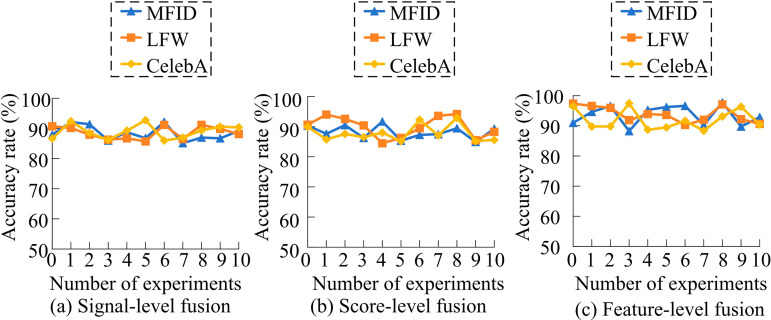
Effects of different fusion schemes on face feature extraction.

In Table 3, the FPR of N0 on different datasets stays between 5.50% and 6.00%, and the accuracy ranges from 90.20% to 93.50%, which is a moderate performance. The performance of the method is improved to varying degrees when either N1 or N2 is added alone. The method performance is optimal when combining N1 and N2. The FPR is the lowest, between 2.50% and 3.50%, and the accuracy is between 93.80% and 96.30%. It is overall better than the performance of the benchmark ResNet18 with the addition of N1 or N2 alone. This shows that the combination of CD and SD attention mechanism for ResNet18 can substantially improve the face feature extraction. On this basis, the effects of different information fusion schemes on face feature extraction are compared. The fusion schemes are signal-level, score-level and feature-level fusion. The results are shown in Fig 11.

**Fig 12:**
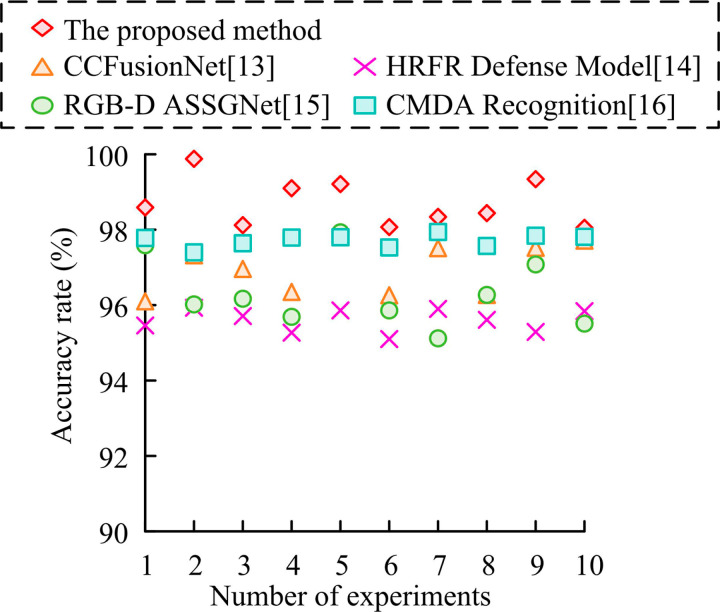
Comparison of facial feature extraction effect.

In Fig 11(a), (b), and (c), among the three fusion schemes, the signal-level fusion is the weakest, with the accuracy hovering around 90% in the three datasets, which is lower than the other two schemes. Fraction-level fusion has slightly improved accuracy and performs better on the LFW dataset, with the highest accuracy close to 95%, but with poorer stability. In contrast, feature-level fusion performs the best, with its accuracy remaining above 90% on all datasets with minimal fluctuations. Moreover, it makes full use of the complementary nature of RGB and depth information. Therefore, feature-level fusion is the most effective fusion strategy among the three schemes. Finally, the study compares the feature-level fusion-supported face feature extraction algorithm based on the improved ResNet18 with the more advanced face recognition technologies presented in references [13–15], and [16]. These technologies are respectively named Capsule-Convolution Fusion Network (CCFusionNet), High-Resolution Face Reconstruction Defense Model (HRFR Defense Model), RGB-D Assisted Segmentation-Guided Recognition Network (RGB-D ASSGNet), and Cross-Modal Domain Alignment Recognition Method (CMDA Recognition). The specific results are shown in Fig 12.

In Fig 12, the accuracy of the study’s proposed method exceeds 98.34% in all experiments and reaches a maximum of 99.64%, showing great stability and accuracy. CCFusionNet [13] has the next best performance, with accuracy ranging from 96.27% to 97.88%. Its performance is relatively stable but still lower than the proposed method. The accuracy of HRFR Defense Model [14] ranges from 95.14% to 95.89%, which is the lowest among all the reference methods. RGB-D ASSGNet [15] performs better in some experiments, with accuracy between 95.29% and 97.35%, but fluctuates more and is less stable than the proposed method. CMDA Recognition [16] performs slightly better than CCFusionNet [13], but there is still a significant gap with the proposed method, with accuracy ranging from 97.2% to 97.98%. In summary, the face feature extraction algorithm proposed in the study performs well in all the experiments and outperforms other advanced methods in the references.

Finally, to verify the generalization ability of the proposed ResNet18 model based on the mixed-domain attention mechanism on the data in the untrained domain and ensure that the model can maintain a high RA in the face of data in extreme environments, a data set completely independent of the training set is used in this study. The Extreme Face Test Dataset (EFTD). The data includes High Intensity Lighting, Occluded Face, Extreme Side Angle, and Different Skin Tones. The sample number of each type is about 500, a total of 2000. The experimental results are shown in Table 4.

**Table 4:** Results of generalization ability verification.

Data Category	EFTD Accuracy (%)	EFTD FPR (%)	EFTD FNR (%)
High Intensity Lighting	97.82%	3.51%	2.18%
Occluded Face	98.67%	4.02%	3.27%
Extreme Side Angle	98.05%	3.67%	2.96%
Different Skin Tones	97.99%	4.15%	3.03%

In Table 4, the proposed ResNet18 model based on the mixed-domain attention mechanism shows good generalization ability in EFTD data sets. The RA rate in all kinds of extreme scenarios exceeds 97.82%. In scenarios characterised by intense illumination, occlusion, extreme lateral views and SC diversity, the model demonstrates the capacity to maintain low PR and FNR, particularly in contexts of occlusion and light variation. This illustrates the model’s resilience and adaptability in challenging environments, and validates the efficacy of mixed-domain attention mechanism in enhancing face feature extraction.

Based on the original EFTD test set, three types of image noise, Gaussian noise, salt and pepper noise, and motion blur, are added to each image. The images with each type of noise and different intensity are tested. The results are shown in Table 5.

**Table 5:** Anti-noise test results.

Noise type	Noise Level	EFTD accuracy (%)	EFTD FPR (%)	EFTD FNR (%)
No noise	/	97.98	4.05	2.22
Gaussian noise	0.01	96.40	4.88	3.15
	0.03	94.72	4.45	3.83
	0.05	92.89	6.02	4.61
Salt-and-pepper noise	0.01	96.12	4.68	3.23
	0.03	93.91	5.97	4.02
	0.05	90.78	6.83	5.15
Motion blur	3	95.87	4.58	3.32
	5	93.20	5.73	4.07
	7	91.56	6.40	4.93

**Fig 13:**
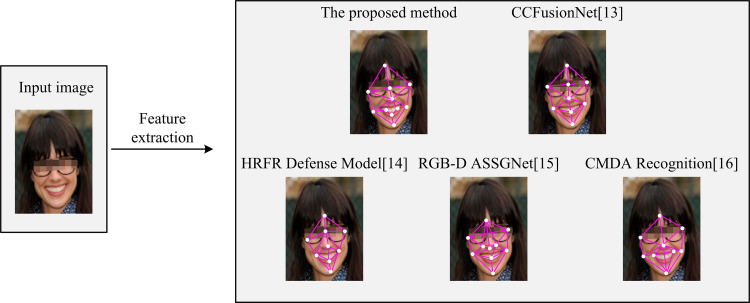
Face feature extraction results.

In Table 5, the proposed ResNet18 model based on the mixed-domain attention mechanism maintains a high RA under different types and intensivities of noise, and shows strong anti-noise performance. Under mild noise conditions (Gaussian noise 0.01, 0.01, 3), the accuracy of the model is more than 96%, and the FPR and FNR are relatively low. However, with the increase of noise intensity, the accuracy of the model decreases somewhat under high intensity salt-and-pepper noise (0.05) and motion blur (7), but it can still remain above 90%. The results show that the model still has strong robustness under high noise environment. The final face feature extraction results are shown in Fig 13.

In Fig 13, the proposed method is more effective in face feature recognition. The methods of CCFusionNet [13] and HRFR Defense Model [14] extract fewer feature points, while the methods of RGB-D ASSGNet [15] and CMDA Recognition [16] have relatively more feature points. However, compared with the methods proposed in this study, the contour drawn is less accurate. Therefore, the proposed method can be used as an effective face feature extraction method.

## 5. Discussion and conclusion

Aiming at the problems of low RA and insufficient generalization ability of face recognition technology in complex environments, the study proposed an improved ResNet18face feature extraction algorithm based on the attention mechanism of hybrid domain. First, the AdaBoost algorithm was improved to increase the accuracy of FD, and the face region was accurately localized by combining SC detection and depth information. Then, the ability to extract features from FIs was further enhanced by introducing the attention mechanism of CD and SD in the ResNet18 network. The outcomes indicated that the RA of the proposed method on MFID, LFW, and CelebA datasets were over 98.34% and up to 99.64%, while the accuracy of the methods in the references was generally lower than 98.00%. In addition, the proposed method also performed well on FPR and FNR. Specifically, after combining the CD and the attention mechanism of the SD, the FPR was as low as 2.50%, which was significantly lower than the FPR of the other methods. It showed that there were clear benefits to the suggested approach in terms of lowering misidentification and enhancing recognition stability. In general, the improved ResNet18 algorithm proposed in this study effectively improves the ability of face feature extraction by introducing mixed-domain attention mechanism.

However, with the introduction of the attention mechanism, the improved ResNet18 model introduces additional computational overhead. First, the attention module increases the number of parameters in the model, causing the storage requirements of the overall model to rise. Second, CD and SD require additional convolution and pooling operations, resulting in floating-point operations (FLOPs) per residual module, increasing computational complexity. In addition, the introduction of attention mechanisms may extend the inference time of the model, affecting the response speed in real-time applications. Finally, the attention module requires additional memory to store intermediate feature maps and weight parameters.

Therefore, future research will focus on optimizing computational overhead and enhancing computational efficiency by designing more efficient attention mechanisms or adopting lightweight models to reduce the number of parameters and floating-point operations. Additionally, attention will be given to the model’s inference speed to meet the demands of real-time applications. Moreover, plans include adding more feature distribution visualizations, such as t-SNE and UMAP, to more comprehensively demonstrate the model’s feature learning effectiveness under various conditions and to compare them with baseline methods, thereby further validating the generalization capabilities and robustness of the proposed method.

## Supporting information

S1 Data Minimal data set. **Figure 1.** No data in Figure 1. **Figure 2.** No data in Figure 2. **Figure 3.** No data in Figure 3. **Figure 4.** No data in Figure 4. **Figure 5.** No data in Figure 5. **Figure 6.** No data in Figure 6. **Figure 7.** No data in Figure 7. **Figure 8.** Verification results of improved AdaBoost algorithm. **Figure 9.** Comparison of face recognition technologies. **Figure 10.** Experimental results of baseline network comparison. **Figure 11.** Experimental results of baseline network comparison. **Figure 12.** Comparison of facial feature extraction effect.
(DOC)
