# Supplementary material for: ResNet18 facial feature extraction algorithm improved based on hybrid domain attention mechanism
Source: PLoS One. 2025 Mar 19;20(3):e0319921. doi: 10.1371/journal.pone.0319921 (PMC11922290; doi:10.1371/journal.pone.0319921)
Supplement: S1 Data — Figure 1. No data in Figure 1. Figure 2. No data in Figure 2. Figure 3. No data in Figure 3. Figure 4. No data in Figure 4. Figure 5. No data in Figure 5. Figure 6. No data in Figure 6. Figure 7. No data in Figure 7. Figure 8. Verification results of improved AdaBoost algorithm. Figure 9. Comparison of face recognition technologies. Figure 10. Experimental results of baseline network comparison. Figure 11. Experimental results of baseline network comparison. Figure 12. Comparison of facial feature extraction effect. (DOC) [file pone.0319921.s001.doc]

**No data in Figure 1**

**No data in Figure 2**

**No data in Figure 3**

**No data in Figure 4**

**No data in Figure 5**

**No data in Figure 6**

**No data in Figure 7**

**Figure 8. Verification results of improved AdaBoost algorithm**

| (a) Training set | | | | | |
| --- | --- | --- | --- | --- | --- |
| Number of weak classifiers | 0 | 25 | 50 | 75 | 100 |
| Standard AdaBoost | 75.46% | 90.34% | 95.79% | 96.21% | 96.15% |
| Improve AdaBoost | 75.38% | 97.67% | 98.41% | 99.62% | 99.64% |
| (b) Test set | | | | | |
| Number of weak classifiers | 0 | 25 | 50 | 75 | 100 |
| Standard AdaBoost | 75.85% | 86.82% | 95.21% | 96.03% | 96.22% |
| Improve AdaBoost | 75.99% | 89.64% | 96.97% | 97.20% | 97.86% |

Figure 9 Comparison of face recognition technologies

| Method | Accuracy | Recall | FPR | FNR | IoU |
| --- | --- | --- | --- | --- | --- |
| The proposed method | 95.48% | 93.84% | 3.16% | 6.28% | 82.94% |
| Reference[8] | 93.27% | 90.21% | 4.15% | 8.01% | 78.58% |
| Reference[9] | 94.18% | 92.19% | 3.98% | 7.63% | 80.36% |
| Reference[10] | 93.17% | 90.38% | 4.82% | 8.12% | 77.41% |
| Reference[11] | 95.11% | 92.94% | 3.55% | 6.96% | 79.27% |

Figure 10 Experimental results of baseline network comparison

| (a) Comparison of accuracy (%) | | | | | | |
| --- | --- | --- | --- | --- | --- | --- |
| Epoch | 0 | 20 | 40 | 60 | 80 | 100 |
| VGG16 | 0 | 38.64 | 70.12 | 82.34 | 87.94 | 89.38 |
| DenseNet121 | 0 | 63.15 | 89.46 | 94.31 | 95.06 | 95.17 |
| ResNet18 | 0 | 63.25 | 88.78 | 94.62 | 95.18 | 95.34 |
| (b) Comparison of time (ms) | | | | | | |
| Epoch | 0 | 20 | 40 | 60 | 80 | 100 |
| VGG16 | 25.00 | 13.31 | 9.48 | 8.64 | 8.31 | 8.43 |
| DenseNet121 | 25.00 | 20.11 | 16.31 | 16.28 | 16.04 | 16.45 |
| ResNet18 | 25.00 | 6.14 | 5.21 | 5.03 | 5.13 | 5.06 |

Figure 11 Experimental results of baseline network comparison

| (a) Signal-level fusion | | | | | | | | | | | |
| --- | --- | --- | --- | --- | --- | --- | --- | --- | --- | --- | --- |
| Experiment Number | 0 | 1 | 2 | 3 | 4 | 5 | 6 | 7 | 8 | 9 | 10 |
| MFID | 89.45% | 92.14% | 92.08% | 88.33% | 88.34% | 88.94% | 92.16% | 86.91% | 87.18% | 88.09% | 89.42% |
| LFW | 91.33% | 90.46% | 88.62% | 87.34% | 88.48% | 88.69% | 92.13% | 89.74% | 91.97% | 90.63% | 88.47% |
| CelebA | 88.48% | 92.43% | 89.14% | 88.37% | 88.37% | 90.28% | 88.34% | 88.47% | 90.15% | 91.31% | 91.48% |
| (b) Score-level fusion | | | | | | | | | | | |
| Experiment Number | 0 | 1 | 2 | 3 | 4 | 5 | 6 | 7 | 8 | 9 | 10 |
| MFID | 90.18% | 89.46% | 91.07% | 86.38% | 92.48% | 84.84% | 86.41% | 88.48% | 87.34% | 88.69% | 90.08% |
| LFW | 90.13% | 93.12% | 92.34% | 90.24% | 85.94% | 87.48% | 90.31% | 93.61% | 93.78% | 88.49% | 90.07% |
| CelebA | 90.31% | 86.15% | 87.64% | 88.17% | 88.38% | 88.09% | 90.11% | 89.31% | 91.12% | 90.97% | 91.93% |
| (c) Feature-level fusion | | | | | | | | | | | |
| Experiment Number | 0 | 1 | 2 | 3 | 4 | 5 | 6 | 7 | 8 | 9 | 10 |
| MFID | 95.31% | 97.09% | 91.53% | 97.03% | 96.87% | 92.50% | 96.13% | 92.51% | 96.43% | 91.71% | 90.65% |
| LFW | 91.34% | 95.67% | 97.71% | 95.06% | 92.64% | 96.54% | 91.97% | 92.18% | 95.08% | 97.48% | 93.45% |
| CelebA | 92.32% | 94.19% | 95.61% | 91.19% | 96.18% | 94.85% | 91.01% | 90.42% | 97.81% | 97.45% | 93.46% |

Figure 12. Comparison of facial feature extraction effect

| Experiment Number | 1 | 2 | 3 | 4 | 5 | 6 | 7 | 8 | 9 | 10 |
| --- | --- | --- | --- | --- | --- | --- | --- | --- | --- | --- |
| The proposed method | 98.78% | 99.80% | 99.24% | 99.65% | 99.63% | 99.26% | 99.21% | 99.75% | 98.39% | 98.64% |
| Reference  [12] | 96.08% | 97.26% | 97.11% | 96.98% | 97.80% | 97.2% | 97.06% | 97.63% | 97.22% | 96.52% |
| Reference  [13] | 95.49% | 95.35% | 95.81% | 95.72% | 95.55% | 95.76% | 95.21% | 95.77% | 95.30% | 95.28% |
| Reference  [14] | 97.27% | 97.37% | 96.19% | 97.71% | 95.74% | 98.01% | 96.82% | 96.67% | 96.7% | 97.87% |
| Reference  [15] | 97.52% | 97.53% | 97.27% | 97.95% | 97.75% | 97.57% | 97.68% | 97.27% | 97.98% | 97.37% |
